# Hemodynamic Changes During Cesarean Section Under Spinal Anesthesia in Normotensive and Hypertensive Pregnant Women—A Narrative Review

**DOI:** 10.3390/jcm15062162

**Published:** 2026-03-12

**Authors:** Edyta Zagrodnik, Małgorzata Szczuko, Anna Surówka, Maciej Ziętek

**Affiliations:** 1 Clinical Department of Anesthesiology and Intensive Care of Adults and Children, Pomeranian Medical University, 71-460 Szczecin, Poland; edyta.zagrodnik@pum.edu.pl; 2 Department of Bromatology and Nutritional Diagnostics, Pomeranian Medical University, 71-460 Szczecin, Poland; malgorzata.szczuko@pum.edu.pl; 3 Department of Plastic, Endocrine and General Surgery, Pomeranian Medical University, 71-460 Szczecin, Poland; anna.surowka@pum.edu.pl; 4 Department of General Pharmacology and Pharmacoeconomics, Pomeranian Medical University, 71-460 Szczecin, Poland

**Keywords:** pregnancy, cesarean section, hypertension, preeclampsia, hemodynamics

## Abstract

Data on cardiac and hemodynamic parameters associated with preeclampsia (PE), particularly changes occurring in the immediate perioperative period, remain scarce. These changes are clinically important for the management of patients with severe PE or underlying cardiac dysfunction. Maternal hemodynamics undergo substantial alterations during cesarean section (CS) as a result of sympathetic blockade induced by spinal anesthesia, the vasodilatory effects of general anesthetics, and changes in blood flow related to aortocaval compression in the supine position and during delivery. Massive hemorrhage represents an additional factor contributing to these alterations. In routine clinical practice, maternal heart rate (HR) and blood pressure (BP) are monitored to assess circulatory status. However, a more precise evaluation can be achieved by measuring stroke volume (SV), cardiac output (CO), and systemic vascular resistance (SVR). These parameters are particularly relevant in cases of severe hemorrhage or hypertension, as they may facilitate targeted hemodynamic management. Overall, hemodynamic responses to cesarean delivery under spinal anesthesia appear to differ between normotensive and hypertensive pregnancies. Normotensive parturients seem to be more susceptible to pronounced hypotension following sympathetic blockade, whereas hypertensive disorders of pregnancy are associated with altered vascular reactivity and modified intraoperative hemodynamic responses. Nevertheless, interpretation of these findings remains limited by the heterogeneity of the available studies and the lack of quantitative evidence synthesis.

## 1. Introduction

Spinal anesthesia is the standard anesthetic technique for cesarean delivery. However, because of the rapid redistribution of intravascular volume and marked changes in vascular resistance, maintaining hemodynamic stability in this patient population remains one of the major challenges in modern obstetric anesthesia. The administration of local anesthetics into the subarachnoid space induces extensive sympatholysis through blockade of sympathetic fibers, leading to a rapid decrease in systemic vascular resistance and vasodilation below the level of the block. As a consequence, blood pools in the venous capacitance vessels, markedly reducing venous return to the heart. When preload is critically reduced, the Bezold-Jarisch reflex may be triggered, manifesting as paradoxical bradycardia and profound arterial hypotension. Two key hemodynamic turning points can be distinguished during cesarean section. The first is the induction of spinal anesthesia, particularly the period immediately after establishment of the block, which is associated with a high risk of sudden systemic hypotension. The second is the delivery of the fetus and placenta, which initiates autotransfusion, whereby approximately 500–800 mL of blood from the uteroplacental circulation is rapidly transferred into the systemic circulation. Simultaneously, relief of uterine compression of the inferior vena cava, resulting from resolution of aortocaval compression, leads to a rapid increase in preload and cardiac output.

The classification of hypertension in pregnancy according to the ISSHP 2018 and ACOG 2020 guidelines distinguishes not only the type of hypertension (chronic hypertension, gestational hypertension, and preeclampsia), but also the severity of preeclampsia based on the presence or absence of “severe features.” This distinction directly influences management strategies, the intensity of maternal and fetal monitoring, and decisions regarding the timing of delivery. According to ISSHP 2018 and ACOG 2020, hypertensive disorders of pregnancy are classified on the basis of the onset of hypertension (before or after 20 weeks of gestation), the presence of proteinuria, and the presence of so-called severe features, particularly maternal organ dysfunction and placental abnormalities. The following overview focuses on preeclampsia with and without severe features.

The basic classification of hypertension in pregnancy includes:

1. Chronic hypertension, which is diagnosed when hypertension (RR ≥ 140/90 mm Hg) is found before pregnancy, before the 20th week of pregnancy, or persists for more than 6 weeks after delivery. It can be idiopathic (primary) or secondary (e.g., renal, endocrine) [1].

2. Gestational hypertension, i.e., de novo hypertension (RR ≥ 140/90 mm Hg) occurring after the 20th week of pregnancy in a previously normotensive woman, in which there are no signs of preeclampsia such as proteinuria or symptoms of organ damage or placental disorders. Blood pressure usually normalizes within 6 weeks after delivery.

3. Preeclampsia (de novo or superimposed on chronic hypertension), i.e., hypertension occurring after the 20th week of pregnancy in association with newly developed proteinuria or, regardless of proteinuria, with other features of maternal organ damage or placental dysfunction. It may appear as: de novo preeclampsia (in a previously normotensive pregnant woman), superimposed preeclampsia on chronic hypertension.

The basic requirement for the diagnosis of preeclampsia is RR ≥ 140/90 mm Hg in at least two measurements, taken ≥4 h apart, after the 20th week of pregnancy, and at least one of the following: 1. Proteinuria ≥ 300 mg/day, or protein/creatinine ratio in a single urine sample ≥ 0.3, or positive dipstick test (at least “+”) in two separate tests, if other methods are not available. 2. Thrombocytopenia, with platelet count < 100,000/µL. 3. Liver dysfunction: aminotransferase (AST/ALT) activity > 2× the upper limit of normal, with or without symptoms (epigastric/right upper quadrant pain). 4. Renal impairment: serum creatinine ≥ 1.1 mg/dL (≈97 µmol/L) or a twofold increase in creatinine from baseline with no other cause. 5. Pulmonary edema. 6. Neurological symptoms: refractory headache, visual disturbances (scotoma, double vision, transient blindness), convulsions (eclampsia, if there is no other neurological cause). 7. Placental disorders (signs of placental insufficiency): intrauterine growth restriction (IUGR), abnormal flow in the uterine/umbilical arteries on Doppler examination, intrauterine fetal death without other clear causes, premature placental abruption.

Preeclampsia without severe features

Criteria for hypertension + (most often) proteinuria met, BUT without so-called “severe features,” i.e.,: severe hypertension (see below), impaired liver function with epigastric/right hypochondrial pain, significant renal dysfunction (except for moderate proteinuria), thrombocytopenia < 100,000/µL, neurological symptoms (convulsions, recurrent headaches, visual disturbances), pulmonary edema, severe placental disorders (severe IUGR, critical flow disorders). In practice, this is a “milder” form of preeclampsia, often possible to manage temporarily under observation and with optimized treatment, depending on the gestational age and well-being of the fetus.

Preeclampsia with severe features

ACOG 2020 and ISSHP 2018 define “severe features” as the presence of at least one of the criteria listed below; their presence automatically qualifies the patient for the category of “preeclampsia with severe features,” even in the absence of proteinuria. The criteria for severity (severe features) include: 1. Severe hypertension RR systolic ≥ 160 mm Hg or diastolic ≥ 110 mm Hg, persisting in two measurements taken ≥4 h apart, despite rest and/or treatment. 2. Significant renal impairment (creatinine ≥ 1.1 mg/dL (or ≥97 µmol/L) or a twofold increase from baseline in the absence of other causes of nephropathy). 3. Severe hepatic impairment (AST/ALT activity > 2× the upper limit of normal, often accompanied by constant pain in the epigastrium or right hypochondrium, liver tenderness, or suspected/onset of HELLP syndrome (hemolysis, elevated liver enzymes, low platelet count). 4. Thrombocytopenia (platelet count < 100,000/µL). 5. Neurological symptoms or visual disturbances, severe, persistent headache resistant to analgesic treatment, visual disturbances (photopsia, scotoma, double vision, transient blindness), symptoms of encephalopathy or stroke, convulsions (eclampsia) not explained by any other cause. 6. Clinical or radiological features of acute pulmonary edema. 7. Severe placental/fetal disorders (significant IUGR, abnormal Doppler velocimetry in the umbilical arteries (e.g., absent or reversed end-diastolic flow), placental abruption.

Patients with chronic or pregnancy-induced hypertension, such as that occurring in the context of preeclampsia, are at increased risk of cardiovascular complications. A characteristic feature of hypertensive pregnancies is relative intravascular hypovolemia, resulting from increased vascular permeability and fluid shift into the extravascular space, manifesting as edema. In these patients, the hemodynamic response to sympathetic blockade is markedly accelerated. Severe hypotension is often more difficult to correct pharmacologically and poses a direct threat to uteroplacental perfusion, potentially leading to fetal hypoxia. At the time of autotransfusion following delivery of the fetus, the heart—previously adapted to high vascular resistance, often with features of left ventricular hypertrophy—may be subjected to sudden and massive volume overload. This can precipitate acute left ventricular failure and cardiogenic pulmonary edema. Hypertensive vasculopathy also predisposes individuals to exaggerated pressor responses to pain stimuli or administration of uterotonic agents, such as oxytocin. Sudden blood pressure surges in this population carry a significant risk of cerebrovascular events, including hemorrhagic stroke due to intracranial vessel rupture. For uncomplicated gestational hypertension, delivery is planned between 37 and 39 weeks [2]. In preeclampsia, immediate delivery is recommended after 37 weeks [3], or earlier if severe features such as refractory blood pressure > 160/110 mmHg, multi-organ dysfunction (renal, hepatic, or hematologic), or compromised fetal well-being arise. Hypertensive women face a significantly higher likelihood of cesarean section [4]. Ramos Filho et al. analyzed 36,724 women and reported a significantly higher incidence of cesarean sections in the hypertensive group (60.22%) versus the non-hypertensive group (31.21%) [4]. The calculated odds ratio for cesarean section was 3.34 (95% CI: 3.14–3.55), confirming a greater than threefold increase in risk. These data clearly establish that performing cesarean sections in hypertensive patients represents a significant clinical challenge for both the obstetric and anesthetic teams [4].

Based on the above facts, detailed analysis of the hemodynamic changes in pregnant women undergoing cesarean section is one of the key research areas in modern obstetric anesthesiology and cardiology. The scientific justification for the importance of this topic is based on several fundamental pillars, such as the physiological uniqueness of the perinatal period, the reduction in perinatal mortality and morbidity, the optimization of fetal well-being, the personalization of medicine in high-risk groups based on standard treatment protocols, and the validation of modern monitoring technologies.

## 2. Methods

This work is a narrative review rather than a systematic review; therefore, it was not designed or reported in accordance with the full PRISMA requirements. However, elements of a systematic approach were incorporated, including a structured search strategy, defined eligibility criteria, and a multi-stage literature selection process. No formal protocol was registered.

A narrative review of the literature on hemodynamics during pregnancy and cesarean section was conducted, with particular emphasis on hypertensive disorders of pregnancy (chronic hypertension, gestational hypertension, and preeclampsia) and the risk of hypotension following regional anesthesia.

The literature search was performed in the international databases MEDLINE, Embase, PubMed, Cochrane Library, and Scopus. Combinations of keywords and MeSH terms were used, including “pregnancy,” “caesarean section,” “hypertension,” “preeclampsia,” “hypotension,” and “hemodynamics,” connected by logical operators (AND/OR). Searches were limited to English-language full-text articles. In this review, screening at the title and abstract stage was limited to assessing thematic relevance, article type, language, and the presence of sufficient methodological information to justify full-text evaluation. We did not perform a formal risk-of-bias assessment at this stage, and no studies were excluded solely on the basis of a presumed high risk of bias inferred from the title or abstract alone. Any concerns regarding methodological limitations were considered descriptively during the full-text review, in line with the narrative nature of this review. To avoid overinterpretation, the manuscript has been revised to remove wording that could imply that a formal or definitive bias assessment was conducted during abstract screening.

Records were independently screened by at least two reviewers at successive stages of selection. Discrepancies were resolved through discussion and consensus, or by involving a third reviewer.

Eligibility criteria. Studies involving pregnant women (both normotensive and with chronic/gestational hypertension or preeclampsia) were included, focusing on the perinatal period and cesarean sections performed under regional anesthesia (spinal, combined spinal-epidural, or epidural when hemodynamic parameters were comparable). Eligible studies had to report at least one measurable hemodynamic parameter (e.g., MAP, SBP, DBP, HR, CO, SV, SVR) during induction or perioperative periods.

Exclusion criteria covered duplicate records, non-full-text materials, abstracts, letters, small case series (<15 patients), and studies lacking hemodynamic data or appropriate populations. After screening and full-text assessment, studies meeting the inclusion criteria and deemed methodologically sound were retained for qualitative synthesis and discussion.

## 3. Results

The 29 studies included in this review were predominantly observational, with randomized controlled trials constituting a smaller proportion of the available evidence. The most frequently analyzed populations were normotensive pregnant women and women with preeclampsia undergoing cesarean section under spinal anesthesia, whereas studies involving chronic or gestational hypertension without preeclampsia were less numerous. Monitoring methodologies were heterogeneous and included standard noninvasive blood pressure assessment, impedance cardiography, continuous noninvasive hemodynamic monitoring, arterial pulse waveform analysis, and echocardiographic techniques. The hemodynamic parameters most commonly assessed were systolic, diastolic, and mean arterial pressure, heart rate, cardiac output, cardiac index, stroke volume or stroke index, and systemic vascular resistance or systemic vascular resistance index. Across the included studies, women with preeclampsia generally exhibited smaller decreases in blood pressure after spinal anesthesia, lower vasopressor requirements, and a predominantly high-resistance hemodynamic profile compared with normotensive parturients. By contrast, findings related to cardiac output were more variable. The evidence regarding chronic hypertension without preeclampsia was more limited and suggested an intermediate profile between normotensive pregnancy and preeclampsia.

The included studies showed consistent differences in the hemodynamic response to spinal anesthesia during cesarean section between normotensive women and those with hypertensive disorders of pregnancy, especially preeclampsia. Overall, women with preeclampsia demonstrated smaller decreases in systolic, diastolic, and mean arterial pressure after spinal anesthesia than normotensive controls, indicating a lower incidence and lower severity of post-spinal hypotension. This finding was supported by the comparative studies summarized in Table 1. In particular, one prospective cohort study reported clinically significant hypotension in 47% of women with preeclampsia compared with 74% of normotensive parturients, while other studies likewise showed less pronounced blood pressure reduction and lower vasopressor requirements in the preeclamptic groups.

The synthesis further showed that preeclampsia was associated with a distinct hemodynamic profile characterized by higher vascular resistance and higher mean arterial pressure, but lower flow-related indices such as stroke index and cardiac index. These findings support the concept that, compared with healthy pregnancy, preeclampsia is a more vasoconstricted and less hemodynamically adaptable state during cesarean delivery under spinal anesthesia. Results concerning cardiac output were less uniform than those for arterial pressure. Some studies showed that cardiac output remained relatively stable or changed only minimally after spinal anesthesia in severe preeclampsia, whereas others demonstrated reductions in cardiac output and stroke volume, suggesting that a smaller fall in blood pressure does not necessarily indicate preserved overall maternal hemodynamic stability. The included evidence also showed differences in vasopressor requirements and treatment effects. Phenylephrine effectively restored mean arterial pressure, but did not consistently increase maternal cardiac output, and the required dose appeared to be lower in women with preeclampsia than in normotensive patients. In the randomized study comparing ephedrine with phenylephrine in preeclamptic patients, both drugs had comparable effects on neonatal acid-base parameters, with no significant differences in umbilical cord pH or lactate concentrations. In low-risk pregnancies, randomized evidence comparing phenylephrine with norepinephrine showed similar fetal cardiac and neonatal safety profiles. Within the broader synthesis, norepinephrine appeared to offer the advantage of better preservation of maternal heart rate and cardiac output, although the evidence in hypertensive pregnancy remained more limited than for phenylephrine. Another recurrent finding was that delivery of the fetus and placenta represented a major hemodynamic transition point. In both healthy and hypertensive pregnancies, this phase was associated with increased venous return and a rise in cardiac output; however, in women with preeclampsia, the increase in cardiac index appeared to depend more on heart rate than on stroke volume augmentation, which may reflect impaired ventricular adaptation to acute volume load. Evidence regarding chronic hypertension without preeclampsia was less extensive, but the available data suggested an intermediate pattern between normotensive pregnancy and preeclampsia. The most pronounced perioperative changes in blood pressure, systemic vascular resistance, and cardiac index were observed in severe preeclampsia, supporting the view that vascular dysfunction is the dominant determinant of the intraoperative hemodynamic course in this subgroup. Overall, the narrative synthesis identified three principal findings. First, spinal anesthesia–induced hypotension is generally less frequent and less severe in preeclamptic than in normotensive pregnancies; second, preeclampsia is characterized by a predominantly high-resistance circulation with altered flow adaptation; and third, assessment of maternal hemodynamics during cesarean section should not rely solely on blood pressure values, as cardiac output-related parameters may provide additional clinically relevant information in high-risk patients.

## 4. Discussion

The findings of this review should be interpreted in the context of the distinct cardiovascular physiology of normal pregnancy and hypertensive disorders of pregnancy. This section therefore focuses on the physiological and clinical implications of the synthesized evidence rather than repeating the results. In normotensive pregnancy, reduced systemic vascular resistance and increased cardiac output create a circulatory state that is particularly vulnerable to sympathetic blockade. This explains why spinal anesthesia during cesarean section commonly leads to marked hypotension in otherwise healthy parturients and why prompt vasopressor support is often required. In contrast, preeclampsia represents a fundamentally different hemodynamic condition, characterized by endothelial dysfunction, increased vascular tone, impaired venous return, and reduced cardiovascular reserve. Although the fall in blood pressure after spinal anesthesia may be less pronounced in these patients, this should not be interpreted as evidence of hemodynamic stability. The underlying circulation remains abnormally altered and may be less capable of adapting to rapid perioperative changes. An important implication is that blood pressure alone does not fully reflect maternal circulatory status. Restoration of arterial pressure is clinically necessary, but it does not always indicate adequate preservation of cardiac output or uteroplacental perfusion. This is particularly relevant in preeclampsia, where placental blood flow may already be compromised and maternal cardiac adaptation may be limited. The period surrounding fetal and placental delivery appears to be especially important from a hemodynamic perspective. Sudden autotransfusion and increased venous return may be well tolerated in healthy women, but in preeclamptic patients they may expose limited ventricular reserve and increase the risk of fluid intolerance, pulmonary edema, or acute cardiovascular deterioration. These considerations support an individualized anesthetic approach in hypertensive pregnancy. In patients with preeclampsia, management should balance the prevention of hypotension against the risk of hypertensive overshoot and circulatory overload. This helps explain why restrictive fluid administration and careful vasopressor titration are emphasized more strongly in this group than in normotensive women. The interpretation of vasopressor therapy should also extend beyond blood pressure correction alone. Although phenylephrine remains the principal agent used for spinal anesthesia–induced hypotension, preservation of maternal flow parameters may be equally important in selected high-risk patients. For this reason, interest in norepinephrine has increased, particularly in contexts where maintaining heart rate and cardiac output may be advantageous. At the same time, hypertensive disorders of pregnancy should not be treated as a single uniform category. Preeclampsia includes heterogeneous hemodynamic phenotypes, and women with chronic hypertension without preeclampsia may differ substantially from those with severe preeclampsia in vascular reactivity and perioperative cardiovascular behavior. This heterogeneity limits the generalizability of simplified management assumptions. The present review should also be interpreted in light of important methodological limitations. The available evidence is based largely on observational studies with relatively small sample sizes, heterogeneous monitoring techniques, and inconsistent definitions of hypotension. Therefore, the present discussion is intended to provide a clinically oriented interpretation of the available literature rather than definitive guidance. Overall, the main message is that cesarean delivery under spinal anesthesia in hypertensive pregnancy should be understood through a broader hemodynamic perspective, not a blood pressure-only model. The clinical challenge in preeclampsia is not merely whether hypotension occurs, but how altered vascular tone, impaired reserve, and reduced tolerance to rapid volume shifts modify the maternal response to anesthesia.

### 4.1. Hemodynamic Changes During Pregnancy, the Perinatal Period, and the Postpartum Period

Normal pregnancy induces profound physiological hemodynamic changes. The heart rate increases by 15–25% above baseline, commencing in the first trimester, peaking in the third, and returning to pre-pregnancy levels at 10 days after delivery [13]. The plasma volume expands by an average of 40% by the 24th week of pregnancy, while a less substantial 30% increase in red blood cell mass results in dilutional anemia [14]. A rapid, progressive 30% increase in cardiac output occurs during the first and second trimesters, and can reach 45% above pre-pregnancy values by week 24. Cardiac output is a further 15% higher in twin pregnancies. The early increase in CO is driven primarily by increased stroke volume; later in the third trimester, SV declines due to inferior vena cava compression by the gravid uterus, making increased HR the primary driver of CO maintenance [15]. Systolic, diastolic, and mean arterial blood pressure (MAP) all decrease within weeks of conception due to reduced systemic vascular resistance (SVR) [16]. SVR reduction begins by the fifth week, reaching its nadir (35–40% of baseline) in the mid-second trimester before returning to pre-pregnancy levels within two weeks of delivery [16]. This decrease is coupled with a 30% increase in aortic compliance. Healthy pregnancy involves increased sympathetic vasomotor activity and significant activation of the renin–angiotensin–aldosterone axis [16]. During labor, pain and uterine contractions further elevate CO by 20%. The maximum CO occurs immediately after delivery and increases by 60–80% compared to the pre-delivery period. Conversely, natural delivery with epidural anesthesia is associated with decreases in MAP and cardiac index (CI) values [17].

### 4.2. Hemodynamic Changes in Pregnant Women During Cesarean Section

**Figure 1:**
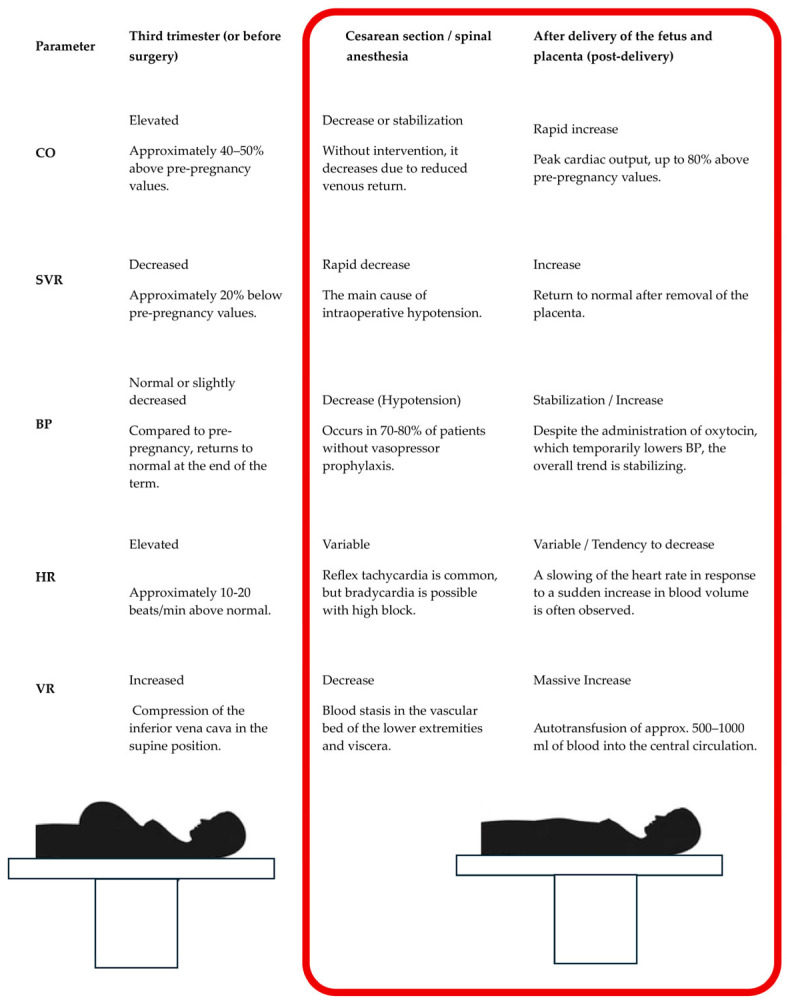
Hemodynamic changes occurring in pregnant women in the third trimester of pregnancy, during a cesarean section under spinal anesthesia, and after delivery of the baby and removal of the placenta [8,27].

Spinal anesthesia, the preferred regional technique for CS, cause rapid peripheral vasodilation leading to a decrease in SVR and the risk of hypotension. Hypotension is defined either by an absolute SBP drop (typically below 80–100 mmHg), a relative drop from baseline, or a combination of both. Crucially, baseline SBP values obtained in the operating room may be artificially elevated due to anxiety [18]. Luther et al. found that the median baseline SBP during elective CS was 15 mmHg higher than the morning reading and 20 mmHg higher than the last antenatal clinic measurement [19]. Hypotension is highly prevalent during planned CS when preventative measures are absent, with the rate of extreme hypotension (SBP < 75% of baseline) reaching 38% [20]. The combination of venous stasis below the block, compressed inferior vena cava, and relative hypovolemia contributes to reduced venous return and reflex tachycardia. Using continuous transthoracic echocardiography, Liao et al. observed that maternal HR decreased by 11.18% and CO decreased by 7.82% post-anesthesia, while SV remained stable [21]. After delivery of the neonate and placenta, SV and CO significantly increased by 21.09% and 22.33%, respectively. End-diastolic volume also increased post-delivery, while end-systolic volume remained unchanged [21]. Preventive strategies include lateral uterine displacement, pre-blockade intravenous fluids (though often debated), and vasopressors (e.g., ephedrine, phenylephrine, norepinephrine) [22]. Phenylephrine, a pure vasoconstrictor, is the drug of choice in healthy pregnant women undergoing CS due to its favorable effect on fetal pH [23]. Noradrenaline (norepinephrine), which possesses a mild *β*-adrenergic effect, achieves an equivalent pressor effect to phenylephrine but causes less reduction in HR and CO [23]. Prophylactic intramuscular phenylephrine has been demonstrated to result in a superior neonatal acid–base balance and more stable maternal hemodynamics, compared to prophylactic intravenous phenylephrine or placebo during spinal anesthesia for elective CS [24]. The position for spinal anesthesia is a contentious topic. Ortiz-Gómez et al. [25] reported no difference in hypotension incidence, vasopressor requirement, or hemodynamic profile when using hyperbaric bupivacaine and fentanyl in the sitting versus lateral position. However, Yun et al. [26] found hypotension to be more severe and longer-lasting with administration in the sitting position during elective CS in healthy women. Thus, the anesthetic position should be one of the factors considered when there is a greater risk to the mother or fetus associated with hypotension [25]. The delivery of the baby and expulsion of the placenta generate significant hemodynamic shifts, including a sudden decrease in abdominal pressure, enhanced venous return from the lower limbs and uterus, and a transient increase in CO. Uteroplacental circulation closure further raises SVR. Under epidural anesthesia for elective CS, significant increases in SV and CO occur after placental delivery [18], which remain elevated until the procedure ends. Similarly, studies during spinal anesthesia for CS have reported increases in MAP and CI [11]. In most cases, early administration of phenylephrine whether via bolus, continuous infusion, or intramuscular injection is widely recommended. Recent evidence suggests that the HR response to pressor drugs serves as a useful surrogate marker for CO [12]. Comparable prophylactic doses of phenylephrine or norepinephrine demonstrate similar effects on fetal HR and fetal CO changes post-spinal anesthesia, with neither vasopressor exhibiting significant adverse effects on fetal circulation or neonatal outcomes [12]. Pre- and post-delivery hemodynamic changes observed in the third trimester during CS under spinal anesthesia are summarized visually in Figure 1.

### 4.3. Hemodynamic Changes in Pregnant Women During Cesarean Section Under Hypertensive Conditions

Hypertension is hemodynamically characterized by two principal types: one dominated by increased vascular resistance, and the other by increased cardiac output. Ohm’s law dictates that blood pressure is the product of cardiac output and total vascular resistance. Therefore, hypertension can represent a spectrum from high CO and low SVR (volume dominance) to low CO and high SVR (resistance dominance) [28]. Hypertension thus results from increased CO, increased SVR, or a combination of both [29]. Preeclampsia (PE) is recognized as a syndrome involving generalized vascular endothelial activation and inflammation, resulting in widespread hemodynamic disruption [30]. A critical distinction of PE’s hemodynamic phenotype is venous system involvement. Doppler studies show that the venous impedance index increases by 20–120% in PE relative to a physiological pregnancy, varying by organ, gestational age, and disease severity [30]. This venous dysfunction compromises venous outflow from internal organs, reducing venous return to the heart, impairing diastolic function, and decreasing CO. The resulting venous stasis and volume overload in the organ parenchyma have major clinical relevance, contributing to impaired renal function (proteinuria), liver dysfunction (elevated transaminase activity), and peripheral edema [28].

Although the review discusses the hemodynamic distinction between early-onset and late-onset preeclampsia, the included studies could not be consistently assigned to these phenotypes. A substantial proportion of the analyzed reports enrolled women described broadly as having preeclampsia or severe preeclampsia, without a separate stratification according to time of disease onset. Moreover, some cohorts likely included both early- and late-onset cases simultaneously, and the diagnostic framing of these entities was not fully uniform across studies, with variability in the use of gestational-age thresholds, severity criteria, and accompanying clinical features. Therefore, although the early-onset phenotype is generally associated with a more high-resistance hemodynamic profile and the late-onset phenotype with a relatively more hyperdynamic pattern, direct study-level attribution was not methodologically robust in the present review.

The following comprehensive table (Table 1) summarizes key findings from studies detailing the contrasting hemodynamic responses between PE patients and normotensive controls during spinal anesthesia for CS.

Cesarean section in pregnancies complicated by PE poses a unique maternal hemodynamic challenge. Pasokpuckdee et al. identified PE as an independent risk factor for an increased CS rate [31]. PE-complicated pregnancy resulting in CS, regardless of severity, is considered a high-risk surgical intervention associated with complications such as obstetric hemorrhage, pulmonary edema, blood transfusions, and prolonged hospitalization [32]. CS in PE is also linked to higher infectious complication rates and postpartum hypertensive crises [27]. A trial of vaginal delivery may be reasonable in selected women with severe preeclampsia, but severe preeclampsia alone should not be considered an automatic indication for cesarean section; however, the route of delivery should remain individualized according to obstetric and fetal conditions [27]. The current evidence base lacks sufficiently robust randomized studies to permit definitive conclusions regarding the superiority of planned cesarean section versus planned vaginal delivery in severe preeclampsia. Although hypotension induced by spinal anesthesia is common, it is often milder in PE patients than in normotensive women. Preeclampsia itself exhibits variable hemodynamic profiles: early onset is typically defined by low CO and high SVR, while late-onset may resemble physiological pregnancy with high CO and reduced peripheral resistance. The PE cardiovascular system displays significant autoregulatory dysfunction. Given that spinal anesthesia risks hypotension and subsequent uteroplacental hypoperfusion, the goal of hemodynamic monitoring during CS is the rapid detection of changes in maternal CO to allow for immediate intervention and protection of uteroplacental perfusion.

Blood pressure responses and risk of hypotension after spinal anesthesia in women with hypertension.

Comparative studies demonstrate that pregnant women with preeclampsia experience smaller decreases in SBP, DBP, and MAP following induction of spinal anesthesia, when compared to normotensive pregnant women. A prospective study of 140 patients found statistically significant differences in the percentage changes in SBP, DBP, and MAP between preeclamptic and normotensive groups, with less severe decreases in blood pressure observed in the former. The incidence of clinically significant hypotension was significantly lower in the preeclamptic group (47%) compared to the normotensive group (74%) (*p* < 0.05), suggesting a reduced risk of severe hypotension in PE patients after spinal anesthesia [6]. The limited ability of the left ventricle in PE women to adapt to volume load during labor and post-CS may contribute to postoperative cardiac deterioration. Thus, PE hemodynamics differ substantially from those observed in healthy pregnancy. Tihtonen et al. reported that women with PE had a higher systemic vascular resistance index (SVRI) and MAP, but a lower Stroke Index (SI) and CI [5]. In another prospective cohort analysis, the incidence of hypotension after spinal anesthesia was significantly lower in patients with severe pre-eclampsia compared to the control group, despite higher baseline blood pressure in the PE group [33]. These observations are underpinned by the increased vascular tone and specific hemodynamic adaptations associated with preeclampsia, including heightened vascular reactivity and reduced susceptibility of the vascular bed to sympathetic blockade-induced vasodilation.

Strategies to reduce the risk of hypotension following spinal anesthesia in pregnant women with hypertension.

The definition of absolute hypotension includes a drop in systolic blood pressure (SBP) below a specified threshold (usually <90 mmHg or <100 mmHg). Relative hypotension, on the other hand, is defined as a drop in SBP below a certain percentage of the baseline value (usually <80% of the baseline value/a drop of >20%). In severe preeclampsia with fetal distress, the choice between ephedrine and phenylephrine bolus for treating spinal hypotension does not independently affect the fetal acid–base balance [8]. Changes in maternal CO have been shown to correlate more closely with uteroplacental blood flow than blood pressure measured non-invasively [34]. Loh et al. [9] confirmed that spinal anesthesia is associated with minimal changes in CO, consistent with other findings [8]. Dyer et al. specifically noted that spinal anesthesia in severe PE caused only clinically insignificant changes in CO [8].

While the administration of phenylephrine effectively restored MAP, it did not increase maternal CO. Manasij et al. indicated that the phenylephrine requirement during CS was significantly higher in the normotensive group than the PE-complicated group [7]. Oxytocin administration causes a transient, significant drop in blood pressure, and increases in HR and CO. Maintaining stable CO during spinal anesthesia is paramount for ensuring adequate uteroplacental perfusion, even in the presence of mild hypotension. Even with the physiological buffering of preeclampsia, hypotension must be prevented due to the high risk posed to both the mother and fetus. Strategies include left uterine displacement to prevent aortocaval compression and improve venous return, as well as judicious preliminary intravenous hydration. Paradoxically, while preeclampsia elevates blood pressure, its maintenance of high vascular tone may mitigate the acute post-anesthesia drop in blood pressure. The official clinical practice guidelines of the American Society of Anesthesiologists (ASA) confirm and systematize the need for careful, restrictive fluid management in women with preeclampsia due to the strict necessity of avoiding pulmonary and cerebral complications. These guidelines also strongly emphasize early intervention with appropriate vasopressors (e.g., phenylephrine) at an adjusted dose rather than aggressive fluid administration [35]. The international consensus on the treatment of hypotension with vasopressors during spinal anesthesia recommends highly cautious fluid administration (cautious co-loading) and limiting rescue fluid boluses (often to a maximum of 500 mL) in patients with preeclampsia [36]. This approach is intended to avoid circulatory overload, which in patients with damaged endothelium can lead to pulmonary edema (Table 2).

The selection of an appropriate vasopressor and its dosing regimen differ fundamentally depending on the patient’s clinical profile.

Phenylephrine

- Healthy pregnancies (elective cesarean delivery): Currently considered the gold standard for the prophylaxis and treatment of spinal-induced hypotension. It effectively restores vascular tone (systemic vascular resistance, SVR), thereby improving arterial blood pressure and fetal umbilical cord acid-base status. Its primary limitation is the induction of reflex bradycardia, which may lead to an undesirable decrease in maternal cardiac output (CO).
- Patients with PE/ Hypertensive Disorders: Phenylephrine remains the vasopressor of choice; however, evidence indicates a necessity for significant dose modification. Patients with PE exhibit a drastically increased sensitivity to vasopressor agents due to endothelial hyperreactivity. The administration of lower doses (e.g., reduced by 50%) is strictly required to mitigate the risk of hypertensive crisis and subsequent pulmonary edema.

Norepinephrine

- Healthy pregnancies (elective cesarean delivery): Increasingly recommended as a first-line alternative. Due to its mild beta-1 adrenergic receptor agonist activity, norepinephrine maintains heart rate (HR) at a more stable level than phenylephrine. This translates into better preservation of global cardiac output (CO) while achieving equivalent efficacy in blood pressure control. Norepinephrine is increasingly supported by data as the first-line drug for hypotension after spinal anesthesia during cesarean section, especially when the goal is to protect cardiac output (CO) rather than simply normalize blood pressure [37]. At the same time, the conclusions of Dyer et al. on “clinically insignificant” changes in CO in severe preeclampsia require a more cautious and nuanced interpretation [8]. Randomized trials and meta-analyses indicate that norepinephrine, while less effective than phenylephrine in maintaining blood pressure, better sustains heart rate and CO and is less likely to cause bradycardia and reactive hypertension [38]. In analyses covering both low-risk and high-risk pregnancies (including preeclampsia), norepinephrine is associated with a comparable incidence of hypotension and similar neonatal outcomes, while also being associated with a lower incidence of nausea, vomiting, and dizziness than phenylephrine [37].

Patients with PE/Hypertensive Disorders: Recent evidence suggests that dilute norepinephrine may be highly beneficial in the setting of PE, as it stabilizes blood pressure without the risk of exacerbated bradycardia (which is frequently observed in patients receiving prior antihypertensive therapy, such as labetalol). Similarly to phenylephrine, the use of reduced prophylactic and rescue doses is absolutely imperative. In patients with preeclampsia, norepinephrine preserves CO better than phenylephrine or ephedrine (higher CO values, less pronounced bradycardia), which translates into more favorable acid-base balance parameters in the newborn (higher umbilical artery pH, lower lactate). Recent reviews and meta-analyses suggest that although phenylephrine remains very effective in reducing episodes of hypotension, norepinephrine is emerging as the optimal alternative, especially in situations where we do not want to excessively reduce CO (e.g., high risk of placental insufficiency, preeclampsia) [39].

Ephedrine

- Healthy pregnancies (elective cesarean delivery): Relegated to a second-line agent due to proven risks of placental transfer and subsequent fetal acidosis. Its use is currently restricted primarily to clinical scenarios where hypotension is accompanied by clinically significant maternal bradycardia.
- Patients with PE/Hypertensive Disorders: In the context of preeclampsia, ephedrine is generally contraindicated. Its mechanism of action, which involves the release of stored endogenous catecholamines, combined with vascular hyperreactivity inherent to PE, renders its effects highly unpredictable. This can precipitate a dangerous, rapid blood pressure overshoot and undesired tachycardia. It is reserved solely as a last resort for severe bradycardia that is unresponsive to anticholinergic agents.

In women with chronic hypertension without preeclampsia, the hemodynamic response to spinal anesthesia should be interpreted more cautiously than in normotensive pregnancy. Although phenylephrine remains the conventional first-line vasopressor for the prevention and treatment of spinal anesthesia-induced hypotension in obstetric anesthesia, its use in patients with chronic hypertension may require more careful titration. This is because increased baseline vascular tone and impaired baroreflex function may predispose such patients to an exaggerated pressor response and excessive blood pressure elevation. Accordingly, the present review does not intend to imply that ephedrine or fluid boluses are generally preferable to phenylephrine in this subgroup. Rather, these interventions may be considered selectively, depending on the individual hemodynamic profile, baseline blood pressure, heart rate, and overall clinical context. In this framework, standard fluid co-loading and prophylactic phenylephrine infusion should be understood as a general reference strategy in obstetric anesthesia, but not as a uniformly applicable approach for all patients with chronic hypertension. Management in this subgroup should therefore remain individualized, with the aim of avoiding both spinal anesthesia-induced hypotension and excessive pressor responses.

Other hemodynamic parameters after spinal anesthesia in pregnant women with hypertension.

In women with PE, preload infusion increased both SI and HR, resulting in a significant increase in CI, whereas in healthy women in labor, only HR increased. Spinal block reduced SVRI in both groups, but CI remained stable. CI increased in both groups at delivery. In uncomplicated pregnancies, both SI and HR contributed to the increase in CI; however, in PE women, the SI was unchanged. Therefore, the CI increase was solely attributed to the increase in HR. The authors inferred that the inability to increase SI in PE women at the time of delivery suggests potential left ventricular dysfunction in adapting to the volume load, raising concern for an increased risk of pulmonary edema [5]. In a non-invasive hemodynamic monitoring study comparing chronic hypertension in preeclamptic and normotensive women during elective CS, it was found that changes in blood pressure and SVR were most pronounced in the context of severe pre-eclampsia, suggesting that vascular pathology dominates the surgical hemodynamic course in this group. Furthermore, more significant changes in SVR and CI were observed in the preeclamptic group compared to both controls and chronic hypertension patients [40].

The significance of differences in hemodynamic parameters after spinal anesthesia in pregnant women with hypertension for clinical practice.

The observed divergence in hemodynamic responses carries profound clinical im-implications. The smaller decrease in blood pressure after spinal anesthesia in gestational hypertensive and preeclamptic patients translates into a lower vasopressor requirement a finding consistently supported in the analyzed studies [6]. However, the significantly higher baseline blood pressure values in preeclamptic patients necessitate meticulous perioperative planning and anesthetic strategy selection to manage the risk of hypertensive crisis [33].

Differences in other hemodynamic parameters, including vascular resistance and flow indices, inform the optimal choice of anesthesia technique and the necessity for advanced intraoperative monitoring. Clinically, this mandates intensive hemodynamic surveillance and early, goal-directed pharmacological interventions to minimize the risk of maternal end-organ damage. While existing evidence strongly supports these hemodynamic differences, the impacts of varying PE severity and different anesthesia techniques (regional vs. general) on cardiovascular dynamics remain insufficiently explored. The limitations regarding study heterogeneity and low population sizes highlight the critical need for further well-designed prospective studies [41].

### 4.4. Hemodynamic Targets

The primary hemodynamic goal is maintaining systolic blood pressure (SBP) at ≥90–100% of the baseline value. In patients with PE, an SBP decrease to below <80% of baseline must be avoided to protect placental perfusion, as should an increase to SBP > 160 mm Hg (leading to risk of stroke). Although hypotension may be less severe in women with severe preeclampsia (due to high levels of endogenous catecholamines and vascular rigidity), any drop in pressure accelerates fetal hypoxia due to the already compromised placental blood flow.

#### 4.4.1. Preeclampsia (PE)

PE presents the most heterogeneous and high-risk hemodynamic profile, characterized by high peripheral resistance (primary mechanism), reduced CO or, conversely, high CO in the hyperkinetic subtype. Key features include relative hypovolemia, hypersensitivity to catecholamines, up-regulation of vasoconstrictors, and endothelial dysfunction. While some patients show minimal hypotensive response due to high SVR, others face a significantly higher risk of a precipitous drop in blood pressure following spinal anesthesia. Furthermore, PE is linked to an elevated risk of pulmonary edema from fluid overload, placental perfusion disturbances, and eclampsia. Acute blood pressure spikes are common during skin incision, fetal extraction, and administration of oxytocin (especially bolus administration, causing initial tachycardia/hypotension followed by hypertension). Management requires meticulous fluid administration (restrictive therapy, cautious co-loading of 500 mL crystalloids), magnesium sulfate (MgSO_4_) for anticonvulsant and vasodilatory effects, and careful vasopressor titration (phenylephrine preferred, but using lower doses of 25–50% of the standard dose to avoid a severe hypertensive crisis). Advanced monitoring is strongly advised.

#### 4.4.2. Chronic Hypertension (Without PE)

In women with chronic or gestational hypertension, elevated SVR is typical but less extreme than in PE, with CO usually normal or slightly reduced. Arterial stiffness and impaired baroreceptor function are common, and they exhibit a lower risk of acute hypotension post-spinal anesthesia due to elevated baseline vascular tone. However, they are at risk for greater blood pressure variability, overshoots with pressors, and severe spikes during high-stimulus events such as general anesthesia induction. Fluid management should be carefully controlled to prevent overload. Phenylephrine carries a risk of excessive pressure increase; thus, ephedrine or balanced fluid boluses are sometimes preferred. Standard fluid co-loading and prophylactic phenylephrine infusion are typically the gold standard for management, with a low threshold for treating drops in blood pressure.

### 4.5. Novel Contributions and Clinical Implications Beyond Previous Comprehensive Reviews

It should be emphasized that this review provides a new, clinically oriented perspective on hemodynamics during cesarean section under spinal anesthesia in normotensive women and women with hypertension/preeclampsia, combining a detailed analysis of CO/SV/SVR changes with practical management strategies, which is not offered by previous reviews [22,23,28]. Compared to Langesæter et al., which mainly describes acute hemodynamic changes (decrease in SVR, effect of sympathetic blockade) in essentially healthy pregnant women, without a systematic division into normotensive and hypertensive populations, this review expands on this approach with a detailed description of two “critical points” in hemodynamics (the period immediately after blockade and the autotransfusion phase after delivery of the fetus and placenta), with a clear link between these phases and the risk of hypotension, volume overload, and left ventricular failure in patients with hypertension/PE [22]. In contrast to the study by Ngan et al. which focuses on the selection of vasopressors (phenylephrine, norepinephrine, ephedrine) and strategies for the prevention of hypotension in healthy pregnant women, with an emphasis on blood pressure and fetal outcomes, our analysis goes further, integrating data on vasopressors with a comparison of phenylephrine requirements in normotensive women and those with PE (lower requirements in PE), discussing that MAP normalization alone does not always mean optimal CO and uteroplacental perfusion, and an indication that lower doses of phenylephrine and more restrictive fluid therapy are preferred in PE to avoid hypertensive crisis and pulmonary edema [23]. Referring to the work of Gyselaers et al., which focuses on the “hemodynamic pathways” of gestational hypertension and PE (high-resistance vs. high-flow phenotype, role of the venous system), but does not refer to them in detail in relation to spinal anesthesia and the course of cesarean section, our review directly translates these concepts into anesthesiological practice, showing that venous dysfunction plays a key role in PE (increased venous impedance index, impaired venous return, limited ability to increase SV) plays a key role in PE, that the response to spinal block and autotransfusion after delivery differs qualitatively between PE and physiological pregnancy (e.g., smaller BP drops, but a higher risk of acute heart failure and pulmonary edema), that the hemodynamic phenotype (high-resistance vs. hyperkinetic) should determine the fluid strategy and the choice/dosage of vasopressors [28].

The present review integrates the hemodynamic and anesthesiological perspectives on cesarean section under spinal anesthesia in normotensive and hypertensive pregnancies into a single narrative synthesis. In particular, it brings together data on differences in the frequency and severity of hypotension, cardiac output dynamics, vascular resistance, and vasopressor requirements between normotensive women and those with preeclampsia or other hypertensive disorders of pregnancy. It also highlights the clinical relevance of interpreting maternal hemodynamic changes beyond blood pressure alone, with particular attention to cardiac output and uteroplacental perfusion.

At the same time, the practical implications discussed in this review should be interpreted with caution. The available evidence remains limited by the predominance of observational studies, heterogeneous monitoring methodologies, variable definitions of hypotension, and insufficient stratification according to hypertensive phenotype, disease severity, or anesthetic technique. Therefore, rather than providing formal management recommendations, this review aims to offer a clinically oriented synthesis of the available literature and to identify areas in which further prospective and methodologically consistent studies are needed.

The limitations inherent to this review on hemodynamics in pregnancy and CS complicated by hypertension include: a lack of sufficient high-quality cohort studies, variability in the hemodynamic parameters measured, differences in measurement methodologies, and population heterogeneity. These challenges hinder the ability to draw definitive, universal conclusions, emphasizing the importance of detailed inclusion criteria and transparent methodological reporting in future research.

## 5. Conclusions and Clinical Considerations

The hemodynamic response to cesarean delivery, particularly under spinal anesthesia, differs between normotensive parturients and those with hypertensive disorders of pregnancy. Normotensive women typically exhibit physiological pregnancy-induced vasodilation and increased cardiac output, making them more susceptible to pronounced hypotension following sympathetic blockade, which usually responds promptly to standard vasopressor therapy. In contrast, patients with hypertensive disorders of pregnancy may present with altered vascular tone and endothelial dysfunction, which can modify intraoperative hemodynamic patterns and responses to anesthesia.

Based on this narrative review, several practical observations relevant to anesthetic management in hypertensive pregnancies may be considered. However, these points should be interpreted with caution. Owing to the absence of quantitative meta-analysis, formal grading of evidence, substantial methodological heterogeneity, and the limited number of large multicenter studies, these observations should not be regarded as formal clinical recommendations.

Phenylephrine remains the primary vasopressor used to manage spinal anesthesia–induced hypotension, while norepinephrine appears to be a promising alternative in selected settings. Available studies also suggest that advanced hemodynamic monitoring may improve the understanding of maternal cardiovascular responses during cesarean delivery, although its routine role has not yet been established.

Future research should focus on large prospective studies and randomized trials employing standardized definitions of hypotension and consistent hemodynamic assessment methods, in order to strengthen the evidence base in this high-risk obstetric population.

## Figures and Tables

**Table 1:** Summary of hemodynamic responses to spinal anesthesia in hypertensive disorders in pregnancy.

Study Citation	(n)	Aim of Study	Study Design	Monitoring Methodology	Study Design	Parameter	Conclusions
[5]	20	PE vs. control	Prospective observational study	Whole-body impedance cardiography	Moderate (Observational)	SI, HR, CI, SVRI, MAP	In patients with preeclampsia (PE), increases in stroke index (SI), heart rate (HR), and cardiac index (CI) were observed following anesthesia. Postoperatively, SI and CI decreased to levels significantly lower than those measured preoperatively.
[6]	140	PE vs. control	Prospective cohort study	Noninvasive blood pressure monitoring	Moderate (Observational)	SBP, DBP, MAP	Hypotension occurred less frequently in patients with PE than in normotensive parturients. Hemodynamic changes following anesthesia were less pronounced in patients with PE.
[7]	100	PE vs. control	Prospective observational study	Noninvasive blood pressure monitoring	Moderate (Observational)	HR, SBP, DBP, MAP	The decline in SBP after spinal anesthesia was smaller in patients with PE. Normotensive patients exhibited a greater and more rapid reduction in MAP. Vasopressor requirements were lower in the PE group.
[8]	15	PE vs. control	Prospective observational study	Arterial pulse waveform analysis via lithium dilution calibration	Moderate (Observational)	MAP, SVRI	CO remained stable following induction of spinal anesthesia. MAP and SVR decreased significantly in the supine position and after oxytocin administration. Phenylephrine effectively increased MAP without exerting a significant effect on CO.
[9]	30	PE vs. control	Prospective observational study	Continuous non-invasive hemodynamic monitoring	Moderate (Observational)	CO, HR, SBP, DBP, MAP	Hypotension following spinal anesthesia occurred in 50% of patients with PE. Spinal anesthesia was associated with significant reductions in SBP, DBP, MAP, CO, and SV. Postoperative hemodynamic parameters did not differ significantly from intraoperative values.
[10]	133	PE ephedrine vs. phenylephrine	Randomized controlled trial (RCT)	Fetal umbilical cord blood gas analysis and maternal blood pressure monitoring	High (RCT)	Umbilical artery base excess, umbilical arterial and venous pH, umbilical lactate concentrations	Ephedrine and phenylephrine exerted comparable effects on neonatal acid–base status. No significant differences were observed in umbilical cord blood pH or lactate concentrations. Lactate concentrations did not differ significantly between groups.
[11]	61	Low-risk pregnancies	Prospective longitudinal study	Noninvasive Cardiac System regional impedance device	Moderate (Observational)	MAP, CO, TPR	Following spinal anesthesia, CO increased without significant changes in MAP or TPR. After delivery, a transient decrease in MAP and TPR was observed, accompanied by a further increase in CO.
[12]	223	Low-risk pregnancies. Phenylephrine vs. norepinephrine	Double blind randomized controlled trial	Fetal echocardiography and maternal noninvasive blood pressure	High (RCT)	CO, HR, SBP, DBP, MAP	Phenylephrine and norepinephrine had similar effects on fetal cardiac output and heart rate. Both agents were safe with respect to fetal circulation and neonatal outcomes.

Abbreviations: PE—Preeclampsia, SI—Stroke Index, HR—Heart Rate, CI—Cardiac Index, SVRI—Systemic Vascular Resistance Index, MAP—Mean Arterial Pressure, CO—Cardiac Output, TPR—Total Peripheral Resistance, SBP—Systolic Blood Pressure, DBP—Diastolic Blood Pressure.

**Table 2:** Therapeutic strategies for hypotension during regional anesthesia in pregnant women with hypertension, depending on phenotype [35,36].

Parameter	Healthy Pregnant Woman/ Chronic or Gestational Hyper-Tension Without PE	Preeclampsia (PE)
Fluid Therapy	Co-loading (10–15 mL/kg)	Restrictive (max 500 mL bolus, cautious co-loading)
Sensitivity to Vasopressors	Standard	Very high (requires dose reduction)
Preferred Vasopressor	Phenylephrine (maintains fetal pH)	Phenylephrine (stabilizes tachycardia), possibly norepinephrine
Main Risk	Hypotension, nausea	Pulmonary edema, hemorrhagic stroke, placental insufficiency

Vasopressor strategies: healthy pregnancies vs. hypertensive disorders/preeclampsia (PE).

## Data Availability

Data sharing is not applicable to this article as no new data were created or analyzed in this study.
